# Regulation of human airway smooth muscle cell migration and relevance to asthma

**DOI:** 10.1186/s12931-017-0640-8

**Published:** 2017-08-16

**Authors:** Brittany Salter, Cara Pray, Katherine Radford, James G. Martin, Parameswaran Nair

**Affiliations:** 1 Firestone Institute for Respiratory Health, St Joseph’s Healthcare and Department of Medicine, 50 Charlton Avenue, East, Hamilton, ON L8N 4A6 Canada; 2 Meakins Christie Laboratories, McGill University, Montreal, QC Canada

**Keywords:** Airway smooth muscle, Migration, Asthma, Cytokines, Remodelling

## Abstract

Airway remodelling is an important feature of asthma pathogenesis. A key structural change inherent in airway remodelling is increased airway smooth muscle mass. There is emerging evidence to suggest that the migration of airway smooth muscle cells may contribute to cellular hyperplasia, and thus increased airway smooth muscle mass. The precise source of these cells remains unknown. Increased airway smooth muscle mass may be collectively due to airway infiltration of myofibroblasts, neighbouring airway smooth muscle cells in the bundle, or circulating hemopoietic progenitor cells. However, the relative contribution of each cell type is not well understood. In addition, although many studies have identified pro and anti-migratory agents of airway smooth muscle cells, whether these agents can impact airway remodelling in the context of human asthma, remains to be elucidated. As such, further research is required to determine the exact mechanism behind airway smooth muscle cell migration within the airways, how much this contributes to airway smooth muscle mass in asthma, and whether attenuating this migration may provide a therapeutic avenue for asthma. In this review article, we will discuss the current evidence with respect to the regulation of airway smooth muscle cell migration in asthma.

## Background

Asthma is a chronic airway disease, characterized by variable bronchoconstriction, airway hyperresponsiveness (AHR), airway inflammation, and airway remodelling. Airway remodelling is a multifaceted process that encompasses several structural changes in the airway wall, including epithelial changes, basement membrane thickening, up-regulation of the extracellular matrix deposition (ECM), an increased number of sub-epithelial myofibroblasts, and increased airway smooth muscle cell (ASMC) mass. Of the aforementioned structural changes, increased ASMC mass is one of the processes with the potential to contribute substantially to the functional consequences of airway remodelling in asthma.

Numerous studies have reported increased ASM mass in fatal [1–10] and non-fatal asthma [11, 12]. However, there is much debate with respect to the exact mechanism responsible for this evident excess accumulation of airway smooth muscle (ASM) mass in asthma. The prevalent view has been that increased ASM mass stems from a combination of hyperplasia and hypertrophy, which is associated with asthma severity and decline in lung function [13–15]. Over the years research has predominantly focused on the contribution of ASMC hyperplasia to increased ASM mass. Although there is no substantial evidence to show that ASMC migration occurs in vivo within the airways, it is possible that ASMC migration is, in part, responsible for the pathogenesis of ASMC hyperplasia and remodelling in asthma.

In particular, it has been proposed that the migration of ASMCs, from the interstitial compartment or from peripheral circulating hemopoetic stem cell populations, could be a possible mechanism to explain increased smooth muscle mass in the airways of asthmatics [16, 17]. The exact source of migrating ASMCs in vivo remains unknown. Gizycki et al., reported that within 24 h of airway allergen challenge, myofibroblasts accumulated in the submucosal compartment of asthmatic lung biopsies. Given that this response occurred more rapidly than the cell cycle duration for fibroblasts, they proposed that the migration of ASMCs to submucosal compartments of the airway wall is a possible mechanism contributing to myofibroblast accumulation [18]. Furthermore, Kaminska et al., demonstrated that in patients with more severe asthma and airflow obstruction, the luminal border of the ASM was closer to the epithelium, compared to those patients who has less severe obstruction, suggesting that these cells contribute to airway remodelling and obstruction [14]. A more recent analysis of intrapulmonary airways has not confirmed this, but has rather shown the opposite suggesting that the remodeling of ASM adjacent to carinae may differ from that at a distance from the carinae [19]. In addition to the local ASMC migration, recent studies have shown that hemopoietic CD34^+^ progenitor cell migrate into ASM bundles from the peripheral circulation, leading us to consider the possibility of bone marrow-derived circulating hemopoietic CD34^+^ progenitor cells, to be another source of excess ASM mass [20]. Despite an increasing interest in the role of ASMCs in asthma, it remains unknown as to how much of the increased ASM mass is due to proliferation or due to airway infiltration of myofibroblasts, neighbouring ASMCs in the bundle, or hemopoietic progenitor cells from the circulation. As such, further research is required to determine the exact mechanism behind ASMC migration within the airways, how much this contributes to ASMC mass in asthma, and whether attenuating this migration may provide a therapeutic avenue for asthma. In this review article, we will discuss the current evidence with respect to the regulation of ASMC migration in asthma.

## Mechanisms of airway smooth muscle cell migration

Cell migration requires highly conserved molecular machinery to coordinate protrusion of the cell anterior and contraction of the posterior, in turn, driving either directed migration (chemotaxis) or non-directed migration (chemokinesis). Cells have extracellular signals that either promote or impede cell movement through interaction with signal transduction pathways of cellular machinery.

For cellular migration to occur, the distinct steps of cell polarization, protrusion, adhesion, traction, and contraction must materialize in a synchronized fashion to ensure controlled migration. A migrating cell must direct filopodia and lamellipodia protrusions, primarily dependent on actin polymerization, and form contacts from the cell to the extracellular environment (ECE) so that the cell may extend, cling to the ECE, and protrude in the required direction while simultaneously contracting the cell posterior.

Essential to migration is the polarization of cells by designating an anterior and posterior of the cell by the localization of proteins. In eukaryotic cells, Cdc42 is a master regulator of cell polarization, active towards the anterior of the cell [21]. Cdc42 regulates location of lamellipodia formation [22], and positions both the microtubule organizing center (MTOC) and Golgi apparatus in front of the nucleus towards the leading edge, facilitating delivery of membrane and associated proteins needed for protrusion [23, 24]. Migrating cells are able to respond to low chemo-attractant gradients through the accumulation of phosphatidylinositol-3 kinase (PI3K) at the leading edge, and phosphatase and tensin homolog (PTEN) along the sides and posterior of the cell [25, 26]. PI3K amplifies chemotactic signals and generates PIP3/Pi(3,4)P2, which are subsequently removed by PTEN along the sides and cell posterior.

Local Rac activation initiates and maintains cellular protrusions. PI3K products activate several Rac exchange factors along the leading edge of the cell [14] and promotes activation of actin polymerization machinery initiating protrusion. Rho family proteins are the foremost regulators of protrusion, regulating lamellipodia, filopodia actin polymerization, and organization of adhesion. Rac, Cdc42, and RhoG are members of the Rho GTPase family that are required for lamellipodia and filopodia protrusion. Although filopodia and lamellapodia are both actin projections, their formation is regulated by different mechanisms. Actin polymerization proteins are regulated by Ca^2+^, PIP2, small G-proteins, and the coordination of several signalling cascades. Directional protrusions are maintained through several feedback loops, including Rac recruiting and activating PI3Ks [22], stabilization of microtubules [23], and recruitment and clustering of integrins at the leading edge [24]. The cell anterior is defined by restriction of PIP3, active Cdc42 and Rac, while the cell posterior is not as well defined beyond PTEN [25]. Actin must also depolymerize, which is mediated by cellular concentration of Ca^2+^. An increase of Ca^2+^ activates gelsolin, leading to interaction between actin and cofilin, which limits actin filament length and allows for an increased turnover of actin [26].

As the cell extends, focal contacts form between the cell membrane and the ECM to stabilize the cell in its new environment. Integrins act as the ECM protein receptors, forming the linkage between cells and the ECM. Cytoskeletal proteins, vinculin, talin, and a-actinin mediate these linkages [27]. The dynamic turnover of integrins is controlled by tyrosine kinases: focal adhesion kinase (FAK) and Src [28]. In addition, microtubules are an essential part of cell migration, playing a role in protrusion through their growth and dynamic instability, but may also contribute to the decomposition of focal contacts [29].

Once a cell protrusion has been stabilized by focal contacts, tractional forces move the posterior portion of the cell forward. Integrins serve as both traction sites and mechanoreceptors, transmitting information regarding the ECM to the cell allowing it to make necessary changes to the dynamics of the cytoplasm [30–33]. The mechanism is based on the balance of actin polymerization, as well as myosin II activity, resulting in stress fiber formation and contraction of the cell posterior [25]. As tension is generated by the moving cell, the physical link between the actin cytoskeleton and integrin breaks, thereby leaving the integrin attached to the ECM [30]. Adhesion regulators, including FAK and Src, work in the rear of the cell, as well as intracellular calcium levels at the cell posterior deconstructing adhesions [25]. Integrins that remain on the cell surface are endocytosed and reused at the cell anterior to continue the migration process [34].

Recent studies by Cleary et al., have shown that ASMC adhesion induced cortactin phosphorylation at Tyr-421, indicating cortactin activation [35]. Phosphorylated cortactin and Pfn-1 were identified on the leading cell edge. β1-intergin was found to be required for recruitment of c-Abl to the cell edge, and that inhibition of actin dynamics impaired spatial distribution of c-Abl. As such, the targeting of c-Abl may pose as a novel target to inhibit ASMC migration.

**Fig. 1:**
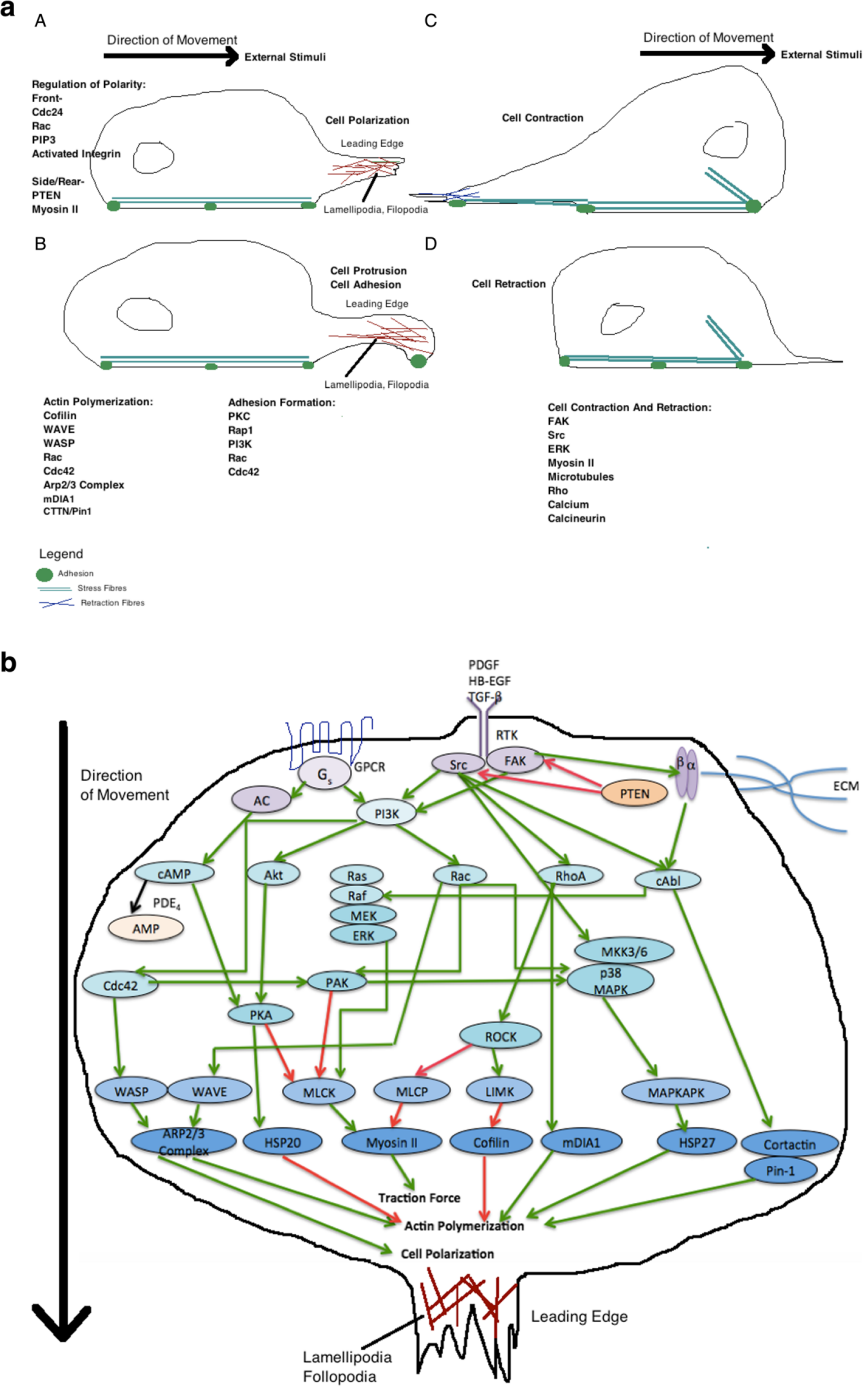
**a:** The process of ASMC migration can be divided into 5 steps: cell polarization, protrustion, adhesion, contraction, and retraction. These steps are controlled by specific signaling proteins, which are modulated by external stimuli. The signaling proteins are described in Fig. 1A. **b:** Cell migration is initiated by activation of receptors such as G protein-coupled receptors (GPCRs), receptor tyrosine kinases (RTK), and integrins, which trigger downstream intracellular signaling, resulting in airway smooth muscle cell (ASMC) migration. An important contributing factor to ASMC migration is actin polymerization, which is a proximal event that propels the leading cell edge towards external stimuli. Various mediators and the extracellular matrix (ECM), for example PDGF, HB-EGF, TGF-β, can activate these membrane receptors. Post-receptor activation, there is downstream induction of trimeric G proteins, Src tyrsoine kinases, phospholipase C, PIP2, c-Abl, and PI3K. This is followed by subsequent activation of signaling proteins (Cdc42, Ras, Rac, Rho, Cortactin, FAK, Akt, PKA, etc), which regulate cell polarization, actin polymerization and traction force. There are various downstream targets that are integral to these processes including the effector proteins mDIA1, WAVE, WASP, ARP2/3 complex. Additional downstream regulators include members of the MAPK family p38 MAPK, ERK, Rho kinase (ROCK), and p21-activated protein kinases (PAK). This results in further phosphorylation of other protein kinases including MAPKAPK, LIMK or phosphatases like MLCP, which then regulate effector proteins that control actin polymerization (HSP20, HSP27, Cortactin, Pin-1, Cofilin) and traction forces (myosin II). Actin polymerization is controlled via complex processes that involve actin branching, actin elongation, and de-branching. This figure is a simplified illustration of the various signaling pathways, which is in reality far more complex then this. These processes are thoroughly reviewed by Tang [127] and Gerthoffer et al. [128]. Red arrows indicate inhibition, green arrows indicate stimulation. Purple circles indicate the most upstream signaling proteins, lightest blue circles indicate effector proteins, including small G proteins, and darker blue circles represent proteins that directly regulate ASMC migration

The specific mechanisms and external signals that govern ASMC migration must also be recognized in order to gain a holistic view of how ASMCs migrate, and subsequently contribute to airway remodelling in asthma pathogenesis. Figure 1 illustrates the current, simplified understanding of ASMC migration signalling mechanisms. ASMCs must adapt to continuous mechanical stress brought on by the breathing motions of the lungs by maintaining the plasticity of the cytoskeleton, and accommodate stresses and strains imposed. Unfortunately, there is little known about the specific external mediators and their signalling pathways involved in human ASMC migration, and more specifically in the context of asthma pathogenesis, thus continued research is required to further elucidate this.

## Promoters of airway smooth muscle cell migration

### Growth factors

Various growth factors, including platelet-derived growth factor (PDGF) and transforming growth factor (TGF)-β have been shown to have a stimulatory effect on ASMC migration in vitro. PDGF has become a well-established promoter of ASMC chemotaxis [16, 17]. We previously reported in an abstract that non-asthmatic smooth muscle cells treated with serum from asthmatics showed increased chemokinesis and chemotaxis towards PDGF and TGF-β compared to untreated cells, which was associated with PI3K activation [36]. PDGF-mediated ASMC migration can be inhibited by the blockade of PI3K signalling, thereby establishing PI3K as a key modulator of PDGF [16, 37]. Furthermore, there is evidence to suggest that the ASMC migratory response elicited by PDGF relies on MAPK activation [38]. TGF-β has been shown to have a synergistic effect with PDGF and facilitate ASMC migration by modifying matrix metalloproteinases (MMPs) and tissue inhibitors of metalloproteinase (TIMPs) balance through the ERK pathway [39]. MMPs degrade the ECM, and together with their inhibitors, TIMPs, they have been suggested to have a role in asthma pathogenesis [40, 41]. Conclusions on how the balance between MMPs and TIMPs influence ASM cell migration have yet to be determined, however the results of Ito et al. suggest that pro-fibrotic growth factors can induce MMPs, and thereby facilitate migration [39]. It has further been demonstrated that PDGF can strongly up-regulate MMP-1 that cleaves a number of collagens and other matrix proteins [40], and when combined with TGF-β results in a synergistic up-regulation of MMP-3 [40]. In addition, PDGF and TGF-β additively enhance expression of TIMP-1 [40].

Heparin-binding epidermal growth factor (HB-EGF) has been implicated in driving ASM mass thickening. Studies have shown that HB-EGF expression to correlate with ASM thickening and that it induces ASMC proliferation in vitro [42]. HB-EGF is also a potent chemotactic factor for various cells including epithelial cells, fibroblasts, and smooth muscle cells [43]. This was expanded upon by Wang et al., who reported that HB-EGF administration accelerated ASM layer thickening in an OVA-induced asthmatic mouse model [44]. They further showed that HB-EGF facilitated ASMC migration in a dose-dependent manner, which was reliant on p38 signalling [44]. Whether HB-EGF influences human ASMC migration remains undetermined. Collectively, these findings demonstrate that growth factors have a potent stimulatory effect on ASMC migration, thereby positioning them as a possible target for attenuating airway remodelling in asthma.

### Lipid mediators

Lipid mediators including leukotrienes and prostaglandins play an important role in asthma pathogenesis. Nair et al., demonstrated that prostaglandin (PG) D_2_ induced ASMC migration, as well as priming migration to PDGF [16]. They suggested that PGD_2_ mediates chemotactic effects on ASMCs through the DP2 receptor expressed on these cells [16]. In addition, they reported that LTE_4_ primed ASMC for migration towards PDGF, which was inhibited following treatment with montelukast and a PI3K inhibitor, suggesting this effect to be mediated through cysteinyl leukotriene receptor (CysLTR) 1 and PI3K pathway signalling [16]. Lastly, they demonstrated the priming effect of LTE_4_ to be attenuated by treatment with lipoxin A_4_ and PGE_2_, thereby proposing that increased cyclic adenosine monophosphate (cAMP) acts as an inhibitory mechanism in ASMC migration [16]. Watanabe et al., examined the expression of LTB_4_ receptors on ASMCs and whether this mediator could effect proliferation and migration [45]. They confirmed the expression of BLT1 and BLT2 on human ASMCs in bronchial tissue and showed that in vitro stimulation with LTB_4_ significantly induced cyclin D1 expression, proliferation, and chemokinesis of human ASMCs. The effect of LTB_4_ on ASMC migration was dependent on BLT1, based on the observation of U75302 attenuating LTB_4_-induced migration. They further showed that LTB_4_induced p42/p44 MAPK and Akt1 phosphorylation. Collectively, given that CysLTR antagonists such as montelukast have been shown to reduce AHR, a possible mechanism for this effect may be due to blockade of ASMC migration, however this warrants further investigation.

### Chemokines

Chemokines are important chemotactic molecules that control trafficking and functions of various leukocytes. The chemotactic chemokine (CC), eotaxin, is increased within the periphery and airways in asthma [46]. Joubert et al., demonstrated that eotaxin can induce ASMC migration in a dose-dependent manner, and that ASMC CCR3 expression is greater in asthmatics compared to healthy controls [47]. In vivo studies have shown epithelial cells and ASMCs produce eotaxin, which may in turn generate an eotaxin gradient, allowing for subsequent migration of ASMC towards smooth muscle bundles and the epithelium [48–50]. This hypothesis is supported by the findings of Pepe et al. that eotaxin production from ASMCs is increased in severe asthmatics compared to healthy controls, which correlated with ASM mass [13].

ASMCs have additionally been shown to express the IL-8 receptors CXCR1 and CXCR2 [50]. The chemotaxis of ASMCs elicited by IL-8 was of a comparable magnitude to PDGF, which is commonly used as a positive chemotactic control [51]. Al-Alwan et al.*,* demonstrated that the CXCL2 and CXCL3, but not CXCL1 can induce significant migration of ASMCs [52]. CXCL2-induced ASMC migration was dependent on p38 MAPK and CXCR2, whereas CXCL3-induced migration was dependent on p38 and ERK1/2 MAPK pathways via CXCR1 and CXCR2 [52]. Lastly, the mast cell-derived chemokine, CCL19, is increased in asthmatics and binds to CCL7, which has been shown to be expressed on ASMCs [53]. It has been thought that the cross-talk between mast cells and ASMCs is mediated, in part, through the CCR7/CCL19, resulting in up-regulated ASMC chemotaxis [54]. Collectively, these findings demonstrate the targeting of chemokines and chemokine receptors on ASMCs may be a novel therapeutic avenue for reducing airway remodelling in asthma, however further investigation is required.

### Cytokines and alarmins

ASMC migration is mediated through a variety of cytokines including IL-13, TNF-α, Th-17-associated cytokines, and alarmins. Parameswaran et al., reported that IL-13 cannot promote a chemotactic nor a chemokinetic response from ASMCs, however it can augment PDGF-primed migration through Src-kinase and leukotriene-dependent pathways, and additionally through up-regulation of PDGF receptors [55]. The precise mechanism appears to be mediated through the IL-4R subunit, and is an aggregate response based on three separate mechanisms including Src-kinase phosphorylation, increase of PDGF receptors, and increase CysLTR expression [55]. With respect to TNF-α, Takeda et al., has reported this pro-inflammatory mediator to increase ASMC migration in a dose-dependent manner [56]. Similar to IL-13, TNF-α cannot directly promote cell migration, but along with its receptors TNFR1 and TNFR2, it is associated with increased production of chemokines IL-8 and RANTES, which in turn promote the migration of ASMCs [57].

Recently, Th17-associated cytokines IL-17A, IL-17F, and IL-22 have also been shown to promote the migration of ASMCs in a dose-dependent manner [57]. IL-17A and IL-17F-induced ASMC migration is dependent on p38 MAPK signalling, whereas IL-22 is dependent on NFκB signalling [57]. Lastly, the pro-inflammatory cytokine, thymic stromal lymphopoietin (TSLP) is highly expressed in ASM bundles from asthma [58] and COPD patients [59], and human ASMCs express its receptor TSLPR [60]. Activation of these cells via TSLPR leads to production of pro-inflammatory mediators IL-6, IL-8, eotaxin-1 [60]. Redhu et al., expanded on these findings to show that TSLP induces ASMC migration, in a STAT3-dependent manner [61]. The aforementioned findings suggest that pro-inflammatory cytokines and alarmins can directly induce ASMC migration and may pose as novel targets for reducing airway remodelling. However, whether other key cytokines, including IL-5 and IL-4, can promote ASMC migration still remains unknown and further studies are required.

### Urokinase

Urokinase, along with its receptor urokinase-type plasminogen activator (uPAR), contributes to the regulation of migratory signal complexes in mammalian cells. Urokinase, alone, does not induce migration of ASMCs [62], but rather increases the effectiveness of cell migration and MAPK activation in the presence of PDGF [62]. It is thought that urokinase enhances ASMC migrational response to PDGF by reorganizing signal transduction molecules [63]. uPar is a glycosylphosphatidylinositol-anchored extracellular protein, lacking a transmembrane and cytoplasmic domain, thus signals are transduced though the formation of other transmembrane proteins, such as integrins [64]. The migration of ASMCs relies on the coordination of membrane proteins and the ECM in the formation of focal adhesions. Carlin et al.*,* demonstrated a model in which urokinase potentiated cell migration and chemotaxis by the destruction of focal adhesion contacts [63]. Co-localization [65] studies and co-immunoprecipitation [66] studies have further demonstrated the association between uPAR and focal adhesions, focal adhesion kinase (FAK) being a central module, physically linking the cytoskeleton to the ECM through internal proteins paxillin and talin and external protein integrins [67]. Urokinase alters the phosphorylation state of focal adhesions by FAK, causing a breakdown of its structure and facilitating cell motility, reinforcing the requirement of a primary stimulus [63]. The mechanism includes the increased association between SHP2 and Src stimulated by urokinase leading to increased phosphorylation of FAK, resulting in proteolysis of FAK and breakdown of membrane domains that impede migration [63]. Although urokinase cannot directly induce ASMC migration, it is clear that urokinase and uPAR may contribute to ASMC migration in asthma. Whether or not targeting urokinase and uPAR can affect airway remodelling, remains to be determined.

### Extracellular matrix

An accumulation of cells and protein in the lamina reticularis has been an established pathogenesis of asthma, characterized by an increase in the amount of ASMCs, collagen, fibronectin, and laminin. It has been demonstrated that collagen V and fibronectin support the migration of ASMCs more than collagens I, and III and laminin, while elastin has no effect [68]. Furthermore, collagen I supports migration in a concentration-dependent manner substantiating the notion of greater actin cytoskeleton polarization of ASMCs on surfaces coated with fibronectin and laminin [69]. The mechanism of ASMC migration by matrix proteins is believed to be triggered by an “outside-inside” signalling event in ASM, leading to an activation of focal adhesion kinase and the 60-kDa c-kinase, followed by a cascade of phosphorylation including paxillin, PI3K, and P38 MAPK, resulting in actin remodelling and chemotaxis [70, 71]. Largely the promotion of ASMC migration via the ECM proteins is dependent on PI3K and SrcK [68]. It is further believed that there is an additional mechanism relating to Src activation through regulation of phosphatidylinositol bisphosphate and intracellular Ca^2+^ levels [72], though the exact mechanism has not yet been described.

### Mechanical (cyclic stretch)

ASMCs are subject to the constant force of stretch and relaxation of the lungs. This cyclical stretch is believed to have an impact on ASMC migration and airway remodelling, ultimately having an influence on asthma symptoms. ASMCs secrete matrix MMPs, a family of zinc dependent proteases that degrade the ECM [73–75]. A study performed by Hasaneen et al. determined that cyclic mechanical strain induced ASMC migration in a MMP-dependent manner, specifically MT1-MMP [73]. The mechanism of cell movement in regards to cyclic stretch may be controlled through the coordination of the cleavage of CD44H by MT1-MMP, the cleavage of γ2 chain of laminin-5, and the processing of the αv subunit of αv β3 integrin [76–80]. The cleavage of CD44H by MT1-MMP appears to support cell migration at the leading edge of the cell through the detachment of CD44H from its ligand [80], while the cleavage of γ2 chain from laminin-5 by MMPs renders laminin-5 active through the exposure of a new functional domain [81], and the processing of αv subunit of αv β3 integrin increases phosphorylation of focal adhesion kinase all enhancing migration. Whether the targeting of cyclic stretch of ASMCs can reduce airway remodelling in human asthma still remains to be determined.

### Viruses

Studies have shown that infections due to human rhinovirus (HRV) during early childhood are associated with a significantly increased risk of developing asthma in subsequent years [82]. Furthermore, evidence has shown that airway remodelling is present in pre-school children before the diagnosis of asthma is established [82]. This suggests that HRV may play a role in promoting ASMC migration. Qureshi et al., demonstrated that serum collected from primary human bronchial epithelial (HBE) cells infected with HRV-16, promoted significant ASMC migration, suggesting that HRV acts as a chemotactic agent for ASMCs [83]. Shariff et al., further showed that HRV-16 infected HBE cells cultured with ASMCs promoted greater ASMC directional migration compared to supernatants from HBE cells exposed to medium, which became maximal at 4 h. Pre-treatment with dexamethasone abolished ASMC chemotaxis [84]. They further showed that HRV-16 induced ASMC migration was most likely due to promotion of CCL5, CXCL7, and CXCL10 secretion from HBE cells, which in turn induced p38, ERK1/2, and JNK pathway activation of ASMCs [85]. These studies demonstrate that HRV infection of airway epithelial cells can promote production of soluble factors that cause directional migration of ASMCs. Whether or not other common respiratory viruses can cause ASMC migration is unknown, and needs to be elucidated further.

## Therapeutic targets and inhibitors of airway smooth muscle cell migration

The emerging hypothesis that ASMC migration is a contributing factor to airway remodelling and subsequent pathogenesis in asthma, justifies the need to develop therapeutic agents to attenuate ASMC migration. Various agents have shown an inhibitory effect on ASMC migration, which may provide useful in the treatment of asthma. Traditional asthma medications, such as beta-agonists and corticosteroids have been shown to reduce ASMC migration in vitro. This raises the question of whether current asthma medication reduces symptoms, in part, by chronically inhibiting ASMC migration.

### Corticosteroids and beta_2_ agonists

Corticosteroids are currently the gold standard treatment for airway inflammation in asthma. Goncharova et al., found that treatment with dexamethasone and fluticasone inhibited PDGF-stimulated ASMC migration, with an observed synergistic inhibitory effect when dexamethasone, fluticasone, and salmeterol were combined [17]. This study provided evidence for corticosteroids acting on ASMCs at the level of gene transcription as cells pre-treated with fluticasone or dexamethasone promoted a significant increase in salmeterol-stimulated CRE activity [17]. They further demonstrated an additive inhibitory effect when glucocorticoids and cAMP elevating agents were combined, but more research is required to explore the cellular mechanism of this interaction [17]. These findings indicate that the current treatment of asthma that routinely combines beta-agonists and corticosteroids is likely to have significant inhibitory effects on migration of ASMCs, thereby attenuating the airway remodelling processes that are dependent on smooth muscle.

### cAMP

The second messenger molecule cAMP is activated by protein kinase A (PKA), and has been shown to have an anti-migratory effect on a variety of cell types including ASMCs, particularly in un-stimulated cells [20, 77]. Intracellular cAMP levels are maintained by the balance of adenylyl cyclase (AC) activity, generating cAMP, and the hydrolysis of cAMP to adenosine monophosphate by membrane bound phosphodiesterases, particularly PDE_4_ in ASMCs [31]. As such, the inhibition of ASMC migration is mediated through increased cAMP production and PKA activity [31]. Goncharova et al., has shown that ASMCs stimulated with Forkskolin, an adenylyl cyclase, resulted in decreased PDGF-induced migration [17]. Other studies have shown that ASMCs exposed to PGE_2_ and beta-agonists, that also increase intracellular cAMP prevent migration of ASMCs [16] through activation of EP prostanoid and beta2-adrenergic receptors respectively [86]; PGE_2_ has a greater inhibitory effect than salmeterol [17]. Furthermore, in cells primed with PDGF, PGE_2_ inhibited ASMC migration, while salmeterol did not have a significant effect on the already pro-migratory signals from PDGF. Cilomolast has also been shown to have an anti-migratory effect of ASMCs through increasing intracellular cAMP by inhibition of type 4 phosphodiesterase (PDE_4_) activity [87], however it did not have an effect on preventing the migration of PDGF primed ASMCs [17]. Other PDE_4_ inhibitors have also been shown to increase intracellular cAMP levels, thereby reducing the migration of ASM under both chemokinetic and chemotactic conditions [17]. Lastly, Kelly et al.*,* reported that treatment of asthmatics with combined therapy of budenoside and formoterol significantly reduced myofibroblast accumulation in the airway submucosa. Although they did not investigate the exact mechanism for this decrease in myofibroblast accumulation, it can be postulated that the use of formoterol increased cAMP levels in these cells, thereby down-regulation their migration to the submucosa [88]. Collectively, these studies demonstrate that increasing cAMP levels in ASMCs, using pharmaceutical agents that modulate AC, PKA, or phosphodiesterase activity may provide a novel therapeutic avenue to reduce ASMC migration and subsequent remodelling in asthma.

### Leptin

A positive correlation has been demonstrated in both cross-sectional studies and prospective studies between obesity and asthma occurrence and severity [86, 89–92]. Leptin, a hormone produced from adipose tissue [93], has been found to be increased in obese patients [94], and even further increased in obese asthmatic patients [95]. Leptin plays a role in regulating the pro-inflammatory effects that are observed in obesity and has recently, due to the correlation, been speculated to participate in the modulation of asthmatic inflammatory responses, including ASMC migration.

In an ovalbumin (OVA) sensitized mouse model, leptin has been shown to increase OVA-induced AHR [96]. The increase in OVA-induced AHR occurred in the absence of an effect of leptin on inflammatory cell influx or Th2 cytokine expression [96]. This suggests that leptin can modulate ASMC function to promote AHR. Nair et al.*,* previously assessed the effect of leptin on human ASMC function [97]. Leptin was shown to inhibit PDGF-induced ASMC migration, proliferation, and IL-13-induced eotaxin production [97]. They further determined that the effects exerted by leptin were mediated through PGE_2_ production from ASMCs, which in turn promotes increased intracellular cAMP [97]. These findings suggest that leptin may be used as a novel therapeutic agent to modulate ASMC migration in asthma, however more studies are needed to determine the exact mechanism of leptin on ASMCs.

### Prostaglandins, lipoxins, sphingosine phosphate inhibitors

Prostaglandins and lipoxins, both products of arachidonic acid metabolism have been shown to reduce ASMC migration. Prostaglandins can inhibit both un-primed PDGF-stimulated and LTE_4_-primed ASMC migration [16, 17]. The reduction in ASMC migration may be mediated through G protein coupled receptors and subsequent stimulation of AC by PGE_2_, followed by PKA activation and accumulation of intracellular cAMP levels [17]. Similar to fibroblast chemotaxis [98] and ASM relaxation [99], the inhibitory effect of PGE_2_ on PDGF-induced ASMC migration has been thought to be mediated through E-prostanoid 1 and 2 (EP1 and EP2) receptor coupled, cAMP-dependent, and PKA mediated pathways [16]. The attenuation of LTE_4_-primed chemotaxis towards PDGF by PGE_2_ may indicate an interaction between EP receptor signalling cascade and CysLTR1 by exerting an inhibitory effect on the Rho-kinase pathway [16]. Lipoxin has also been demonstrated to reduce LTE_4_-primed ASMC migration towards PDGF, despite the fact that ASMCs do not express the lipoxin-A_4_ cognate receptor (ALX) [55]. It is thought that lipoxin competes with cysteinyl leukotrienes at their receptor CysLT1, antagonizing the cysteinyl leukotriene pathway, thereby reducing the migratory response [55]. Lastly, inverse agonism of the sphingosine-1-phosphate (S1P) (a signalling lipid) receptor S1P1 has been shown to inhibit PDGF-stimulated ASMC migration, through the p42/p44 MAPK pathway [100]. S1P1 is normally constitutively active, and a complex between PDGFβ receptor and the S1P1 receptor allows the employment of active G-protein subunits and facilitation of migration [100]. These aforementioned studies suggest that modulation of lipid mediators may pose as novel agents to prevent airway remodelling through reduction in ASMC migration in asthma.

### Retinoic acid

All-trans retinoic acid (ATRA), an active metabolite of vitamin A and its receptor retinoic acid receptor (RAR) have been demonstrated to inhibit PDGF-mediated ASMC migration [101]. There is an additional retinoic acid receptor, retinoid X receptors (RXR), which along with RAR are a part of the superfamily of steroid hormone ligand activated transcription factors [102, 103]. RXR binds only 9-cis retinoic acid (an isomer of ATRA), while RAR binds both ATRA and 9-cis retinoic acid. When RAR and RXR are activated by their ligands the proteins form heterodimers initiating downstream events including activation of gene transcription, activation of other activator proteins, as well as interferes with signalling proteins ERK1/2 [104], PI3K, and Akt [105]. Day et al., demonstrated that ATRA inhibits ASMC migration by disrupting the organization of the cell’s actin cytoskeleton through the PI3K/Akt pathway [101].

Human ASMCs express functional DNA-binding proteins RARα, RARβ, RARγ, RXRα, and RXRβ but not RXRγ [106]. The activation of either RAR or RXR is sufficient to elicit an anti-migratory effect in PDGF-induced ASMCs, supporting the involvement of RAR-RXR heterodimer activation. The inhibition of ASMC migration is thought to be mediated through RARα forming protein-protein interaction with the p85 subunit of PI3K, preventing actin reorganization and migration of the cell, indicating RAR regulation of PI3K signal transduction [101].

### Nuclear hormone receptors

Nuclear hormone Receptors liver X receptor (LXR) and peroxisome proliferator-activated receptor (PPAR) are related in their biological functions, both associated with controlling anti-inflammatory gene expression, and are believed to function in the prevention airway of remodelling, characteristic of asthma. LXRs consist of α and β subtypes, and both are widely expressed in human ASMCs [107]. LXRs control the expression of genes that are related with inflammation homeostasis, and its activation has been demonstrate to inhibit PDGF-stimulated ASMC migration in a concentration-dependent manner [107]. Oxysterols activate LXRs and function by binding LXR-response elements of the target gene, by associating with the obligate heterodimerization partner RXRα [108]. Furthermore, LXRs regulate inflammatory signalling including, pro-inflammatory cytokines like COX-2 [109].

The precise mechanism of how LXR inhibits ASMC migration has yet to be established, but it has been speculated that it is not the Src Kinase, Akt or P38 MAPK pathways [107]. With respect to PPAR receptors, Stephen et al., reported that activation of PPAR receptors expressed on human ASMCs, resulted in decreased migration towards PGDF, an effect independent both of the promotion of PTEN activity, and inhibition of Akt phosphorylation [110]. They determined that the PPAR-mediated inhibition of ASMC migration was through an increase in intracellular cAMP, partly mediated by the induction of COX-2 and production of PGE_2_ [110].

### Src, PI3K, p38 MAPK inhibitors, PTE, c-Abl tyrosine kinase

Various plausible intracellular targets for the inhibition of ASMC migration have been recently identified, including MAPK, Src family tyrosine kinases, PI3K, and Rho-b kinase inhibitors. A 2005 study by Krymskaya et al. demonstrated that Src is necessary and sufficient for human ASMC migration and proliferation [111]. Src protein tyrosine kinase is activated by the integration of differential signalling from GPCR and receptor tyrosine kinase (RTK) and is capable of producing ASMC migration [111]. PDGF signalling promotes RTKs and Src activation, followed by PI3K activation, ultimately resulting in ASMC migration [111]. Additionally, during cell migration it is essential that integrins adhere to the matrix surface, which is mediated through the focal adhesion kinase complex, followed by integrins activating Src kinase (SrcK) and successively inducing the PI3K and MAPK cascades [111]. Increased phosphorylation of SrcK and ASMC migration has been shown to be associated with collagens III, V, and fibronectin, however whether cells were plated on media that strongly promoted, or not as strongly supported migration, the difference in the amount of phosphorylated Src was insignificant [68]. It is clear that although phosphorylation of Src increases chemotaxis, it is not the only regulator of direction ASMC migration [68]. The regulation of phosphatidylinositol bisphosphate and intracellular Ca^2+^ level may be a possible method of increasing chemokinesis and cell motility [113].

PI3K, a chemotactic signal amplifier accumulates at the leading edge of a migrating cell [26] and indirectly activates integrin formation through the activation of PIP3 [114, 115]. In ASMCs, the PI3K pathway has been identified as a key signalling mechanism in balancing the pro-migratory and anti-migratory signals of Rho-guanosine triphosphate (GtP)-ase activating protein (GAP), most notably in regards to PDGF-stimulated chemotaxis [116, 117] The chemotactic response of ASMCs exhibited by LTE_4_ and the CysLTR1 is dependent on the PI3K signalling pathway [16], as well as the chemotaxis modulated by extracellular matrix proteins [68]. ASMC chemotaxis was inhibited by PI3K antagonism. Research has demonstrated that Src stimulation activates PI3K [68, 81], but independent of or parallel to ERK1/2 [118, 119]. A PI3K inhibitor showed a greater reduction of chemotaxis compared to a Rho-kinase inhibitor and p38 MAPK inhibitor, though had a non-significant effect on chemokinesis [16].

The over expression of the PTEN gene reduces the proliferation and migration of ASM cells. Increased PTEN expression reduced the phosphorylation of Akt and FAK, blocked the PI3K/AKT pathways, and ultimately inhibited actin cytoskeleton rearrangement [120].

The Rho/Rho kinase pathways are involved in directional and non-directional cell migration of ASMCs through the regulation of actin cytoskeleton reorganization [113]. Human in vitro studies have shown that ASMC migration induced by PDGF and primed by LTE_4_ were inhibited following blockade of Rho-kinase [16]. It is possible that the inhibitory effect on the Rho-kinase pathway involves interaction between the prostanoid receptor signalling cascade and the CysLTR1, though further study is required [16].

In human ASMCs, blockade of p38^MAPK^ signalling inhibited PDGF-stimulated chemokinesis and LTE_4_-primed PDGF-stimulated chemotaxis [16]. The priming effect was weakened by RhoK, p38^MAPK^ and PI3K inhibitors, thus it is likely that regulation is upstream, at the level of Ras or Src kinase pathways [16]. A study performed with dog tracheal smooth muscle cells that expressed human HSP 27 demonstrated that the inhibition of p38^MAPK^ blocked ASMC migration, and that PDGF, TGFβ, and IL-1β activate the p38^MAPK^ pathway [120]. It was proposed that the activation of p38^MAPK^resulted in the phosphorylation of HSP 27, which may modulate F-actin polymerization, which accounts for the attenuation of migration in p38^MAPK^ inhibited cells [120].

Lastly, Cleary et al., identified a role of c-Abl in AMSC proliferation and contraction [35]. They determined that inhibition of c-Abl can attenuate adhesion-induced cortactin phosphorylation and Pfn-1 localized to the leading edge of ASMCs [35]. It has also been shown that in response to growth factors, Abl can regulate ASMC proliferation [121, 122]. The blockade of c-Abl signalling leads to inhibition of ASMC proliferation induced by growth factors [121, 122]. Expanding on these findings, Cleary et al. has further shown that in an asthmatic mouse model, c-Abl is up-regulated in airway tissue and knockout of c-Abl attenuated airway resistance and ASMC mass [123]. In addition, severe asthmatics were shown to have up-regulated c-Abl expression on ASMCs [123]. These findings suggest that c-Abl plays a role in regulating ASMC migration, and that altered expression of c-Abl is critical for the development of ARH and airway remodelling.

## Putting this in clinical perspective

Currently, other than bronchodilators (beta adrenergic receptor agonists and muscarinic receptor antagonists), there are no approved effective therapies specifically directed at ASM contraction, relaxation or proliferation. The only therapy that may have an effect in decreasing ASM mass is bronchial thermoplasty, which involves the delivery of radiofrequency energy to the airway wall through a bronchoscope [124]. The mechanisms are not known and it is not entirely clear if the effects observed are specifically due to an inhibitory effect on ASM or if they are an indirect effect through other mechanisms such as neuronal injury, epithelial reprogramming, etc. [124]. However, evidence that ASM mass is decreased in both treated and non-treated airways [125] and that the clinical benefits are sustained for as long as 5 years after the procedure [126], suggest that the therapy may indeed be modulating ASM biology. If the cell migratory processes that we have described in this review are relevant, it is not immediately apparent why the airway physiology and symptoms do not worsen over time with gradual accumulation of new myocytes after the local ASM mass is attenuated. It would be critical to do studies in patients who have achieved sustained improvement in AHR and symptoms, particularly to reassess airway myofibroblasts and the migratory properties of mature and immature myocytes and fibromyocytes after an inflammatory stimuli such as an allergen provocation or a controlled respiratory virus infection.

## Conclusion

**Fig. 2:**
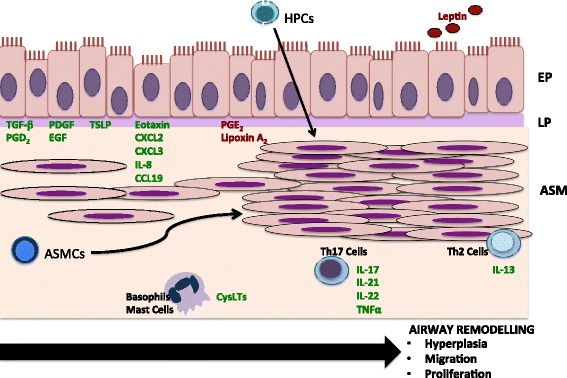
Airway remodeling is an important feature of asthma pathogenesis. An important contribution to airway remodeling is increased ASM mass, which is thought to be brought on by ASMC migration, thereby adding to the local cellular hyperplasia. The source of these ASMCs has been thought to be due to local infiltration of myfibroblasts, neighbouring ASMCs, and circulating HPCs. Various mediators have been identified that influence ASMC migration, which are outlined in this figure. Local pro-inflammatory mediators produced by airway epithelial cells, including TGF-β, PDGF, EGF, PGD_2_, CXCL2, CXCL3, IL-8, eotaxin, TSLP, and CCL19 have been shown to induce ASMC migration, whereas PGE_2_ and Lipoxin A_2_ inhibit ASMC migration. In addition, inflammatory cytokines produced by Th17 and Th2 cells, as well as CysLTs produced by basophils and mast cells, further contribute to ASMC migration. Conversely, systemically circulating leptin inhibits ASMC migration. Abbreviations: EP- epithelium; LP- lamina propria; ASM- Airway Smooth Muscle; ASMCs- Airway Smooth Muscle Cells; HPCs- hemopoetic progenitor cells; CysLTs- cysteinyl leukotrienes

A better understanding of the pathophysiology of asthmatic airway remodelling is crucial to identify new therapeutic avenues for asthma. Emerging evidence has implicated ASMC migration as an important contributing feature to excessive ASM mass in asthmatic airways. Various studies of ASMC migration have defined multiple physiological regulators of cell migration, as well as identified various pharmacological agents with anti-migratory effects (outlined in Fig. 2, Tables 1 and 2). Many of these pharmacological agents act on intracellular signalling pathways and effector proteins that regulate ASMC migration, which may be valuable targets in asthma. Defining the relative contribution of the migration of resident ASMCs versus circulating HPCs to excess ASM mass in asthmatic airways will be important to ascertain in order to develop new targets for asthma treatment. In the future, currently identified targets will need to be evaluated by means of clinical trials to determine the efficacy of these agents in reducing airway remodelling and subsequent symptom reduction in asthma.

**Table 1:** Promoters and Inhibitors of ASMC Migration

Promoters	Inhibitors
PDGF [16, 17, 37, 38]	Lipoxin A_2_ [16]
TGF-β [36, 39]	PGE_2_ [17]
HB-EGF [42–44]	Corticosteroids [17, 88]
PGD_2_ [16]	Beta-Agonists [17, 88]
LTB_4_ [16, 45]	CysLT Receptor Antagonists [16, 86]
Eotaxin [47–50]	Leptin [97]
IL-8 [52]	Retinoic Acid [101]
CXCL2 [52]	PPAR Agonists [107–110]
CXCL3 [52]	Src Inhibitors [111–113]
IL-13 [56]	PI3K Inhibitors [16, 116–119]
TNF-α [56]	P38 Inhibitors [16, 120]
Th-17 [58]	MAPK Inhibitors [16, 120]
Urokinase [62, 63]	PTEN Agonists [120]
uPAR [65–67]	Inverse Agonism of S1P1 [100]
HRV-16 [84–87]	c-Abl Inhibitors [35, 121–123]
LTE_4_ [16]	
CCL19 [53, 54]	
TSLP [61]

**Table 2:** Potential Pharmacological Therapies to Reduce ASMC Migration

Therapies	Summary of findings
Corticosteroids [17, 88] • Fluticasone • Dexamethasone • Budenoside	• Fluticasone and Dexamethasone shown to inhibit PDGF-stimulated ASMC migration in vitro [17]; promote inhibitory effect of Salmeterol on PDGF-stimulated ASMC migration in vitro [17] • Treatment of asthmatics with combined Budenoside and Formoterol reduced myofibrolasts in airway submucosa [88]
Beta-Agonist [17, 88] • Salmeterol • Formoterol	• Inhibition of PDGF-stimulated ASMC migration in vitro [17] • Treatment of asthmatics with combined Budenoside and Formoterol reduced myofibrolasts in airway submucosa [88]
Adenylyl Cyclase Analogue [17] • Forkskolin	• Inhibition of PDF-induced ASMC migration in vitro [17]
PDE_4_ Inhibitor [87] • Cilomolast	• Inhibition of non-PDGF stimulated ASMC migration in vitro [87]
CysLT Receptor Antagonist [16] • Montelukast	• Priming effect of LTE4 on PDGF-induced ASMC migration was inhibited following treatment with Montelukast in vitro [16]
DP_2_/CRTH_2_ Receptor Antagonist [16] • BWA868C	• Inhibition of PGD_2_-stimulated ASMC migration in vitro [16]
PPAR Agonist [110] • Ciglitazone • 15-deoxy-D12, 14-prostaglandin J2 • WY-14643	• Inhibition of PDF-induced ASMC migration in vitro [110]

## Abbreviations

AC: Adenylyl cyclase
AHR: Airway hyperresponsiveness
ALX: Lipoxin-A_4_ cognate receptor
ASM: Airway smooth muscle
ASMCs: Airway smooth muscle cells
ATRA: All-trans retinoic acid
cAMP: Cyclic adenosine monophosphate
CysLTR: Cysteinyl leukotriene receptor
ECE: Extracellular environment
ECM: Extracellular matrix
EP1 and EP2: E-prostanoid 1 and 2
FAK: Focal adhesion kinase
GAP: Guanosine triphosphate-ase activating protein activating protein
GtP: Guanosine triphosphate
HBE: Human bronchial epithelial cells
HB-EGF: Heparin-binding epidermal growth factor
HRV: Human rhinovirus
LXR: Liver X receptor
MMPs: Matrix metalloproteinases
MTOC: Microtubule organizing center
PDE_4_: Phosphodiesterase
PDGF: Platelet-derived growth factor
PG: Prostaglandin
PI3K: Phosphatidylinositol-3 kinase
PIP2: Phosphatidylinositol biphosphate
PKA: Protein kinase A
PPAR: Peroxisome proliferator-activated receptor
PTEN: Phosphatase and tensin homolog
RAR: Retinoic acid receptor
RTK: Receptor tyrosine kinase
RXR: Retinoid X receptors
S1P: Sphingosine-1-phosphate
SrcK: Src kinase
TGF: Transforming growth factor
TIMPs: Tissue inhibitors of metalloproteinase
TSLP: Thymic stromal lymphopoietin
uPAR: Urokinase-type plasminogen activator
